# Evaluation of open search methods based on theoretical mass spectra comparison

**DOI:** 10.1186/s12859-021-03963-6

**Published:** 2021-04-26

**Authors:** Albane Lysiak, Guillaume Fertin, Géraldine Jean, Dominique Tessier

**Affiliations:** 1 CNRS, LS2N, Université de Nantes, 44000 Nantes, France; 2 BIBS Facility, INRAE, 44316 Nantes, France; 3 UR BIA, INRAE, 44316 Nantes, France

**Keywords:** Mass spectrometry, Open Modification Search, Peptide identification, Blind search

## Abstract

**Background:**

Mass spectrometry remains the privileged method to characterize proteins. Nevertheless, most of the spectra generated by an experiment remain unidentified after their analysis, mostly because of the modifications they carry. Open Modification Search (OMS) methods offer a promising answer to this problem. However, assessing the quality of OMS identifications remains a difficult task.

**Methods:**

Aiming at better understanding the relationship between (1) similarity of pairs of spectra provided by OMS methods and (2) relevance of their corresponding peptide sequences, we used a dataset composed of theoretical spectra only, on which we applied two OMS strategies. We also introduced two appropriately defined measures for evaluating the above mentioned spectra/sequence relevance in this context: one is a color classification representing the level of difficulty to retrieve the proper sequence of the peptide that generated the identified spectrum ; the other, called LIPR, is the proportion of common masses, in a given Peptide Spectrum Match (PSM), that represent dissimilar sequences. These two measures were also considered in conjunction with the False Discovery Rate (FDR).

**Results:**

According to our measures, the strategy that selects the best candidate by taking the mass difference between two spectra into account yields better quality results. Besides, although the FDR remains an interesting indicator in OMS methods (as shown by LIPR), it is questionable: indeed, our color classification shows that a non negligible proportion of relevant spectra/sequence interpretations corresponds to PSMs coming from the decoy database.

**Conclusions:**

The three above mentioned measures allowed us to clearly determine which of the two studied OMS strategies outperformed the other, both in terms of number of identifications and of accuracy of these identifications. Even though quality evaluation of PSMs in OMS methods remains challenging, the study of theoretical spectra is a favorable framework for going further in this direction.

## Background

Proteomics is at the core of various studies that aim at understanding the complexity of life. In particular, one of the objectives is to discover all the modifications that can affect proteins, and possibly result in a modulation or a total change of their cellular functions [1, 2]. Mass spectrometry in tandem MS mode (MS/MS) is the most powerful method to identify proteins and characterize their modifications on a large scale. However, it remains frustrating to observe that, in spite of an abundant literature on the subject, most of the spectra generated by this analytical technique—namely, tens of thousands of spectra per hour of analysis—are left unidentified after their analysis by a dedicated software. The reason behind this low rate of identification is likely due to the large proportion of spectra generated from proteins carrying modifications [3]. Software usually infer the identification of an experimental spectrum from its similarity to reference spectra. When a peptide carries a modification, its mass is by nature modified. This mass modification prevents its identification by conventional methods, which compare each experimental spectrum with only a restricted set of reference spectra that approximately share the same mass in order to avoid excessive runtime. Some known modifications can be included in the modeling of reference spectra, but their number must remain low to circumscribe the search space.

In 2015, the study conducted by Chick et al. [4] renewed the interest in so-called Open Modification Search (OMS) methods known since 2005 [5, 6], with the promise of revealing unexpected modifications which would have been lost otherwise, and consequently gaining better rates of spectra identifications. OMS methods compare each experimental spectrum to *all* the reference spectra representing a proteome. Then, whereas conventional methods, by definition, try to identify pairs of spectra (experimental vs reference) that are supposed to represent the *same* chemical compound (i.e., an ideal reference spectrum is matched to its imperfect experimental counterpart), OMS methods allow matches between similar spectra that represent *distinct* chemical compounds with unequal masses. As a result, this comparison produces a list of PSMs (Peptide Spectrum Matches) per experimental spectrum, and a non-zero mass difference $\Delta m$ between the experimental spectrum and its associated peptide is assumed to be due to one or several modifications that differentiate them. Most of the time, only one PSM per experimental spectrum is reported: each experimental spectrum is associated to the most similar reference spectrum. Many scores exist to evaluate the similarity between two spectra, which all take into account, at a certain level, the number of peaks (or, equivalently, of masses) that are shared by the two spectra, a number called shared peaks count (SPC). Once the complete list of PSMs is sorted by score, a threshold based on a measure of statistical significance determines which PSMs are validated according to a false discovery rate (FDR) [7].

**Fig. 1:**
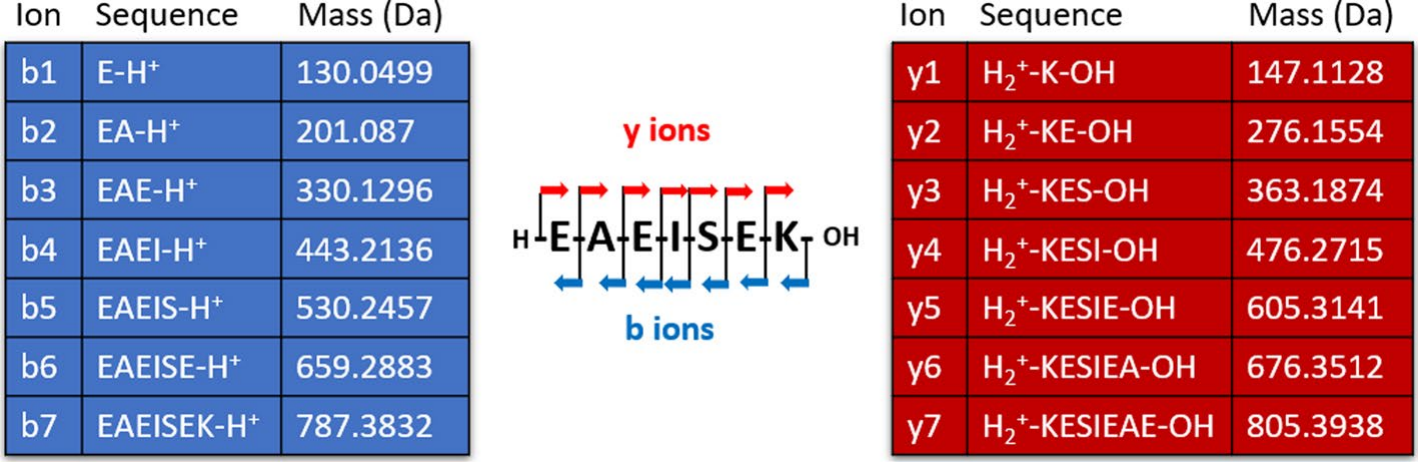
Example of in silico generation of a theoretical spectrum. The theoretical spectrum of peptide EAEISEK is composed of masses of the two main types of ions obtained by mass spectrometry: the *b* series (blue table) and *y* series (red table). A *b*-ion contains the N-terminal part of the peptide and is numbered according to the peptide sequence, from left to right. A *y*-ion contains the C-terminal part of the peptide and is numbered backwards according to the peptide sequence, from right to left. Masses are given in Daltons

Since 2015, several very rapid OMS methods have been developed. For a few of them, the reference spectra come from consensus templates already observed, identified and stored in spectral librairies [8–11]; for others, the reference spectra, so-called theoretical spectra in that case, are generated from a protein database by simulating an ideal fragmentation of peptides (see Fig. 1) [12–14].

**Fig. 2:**
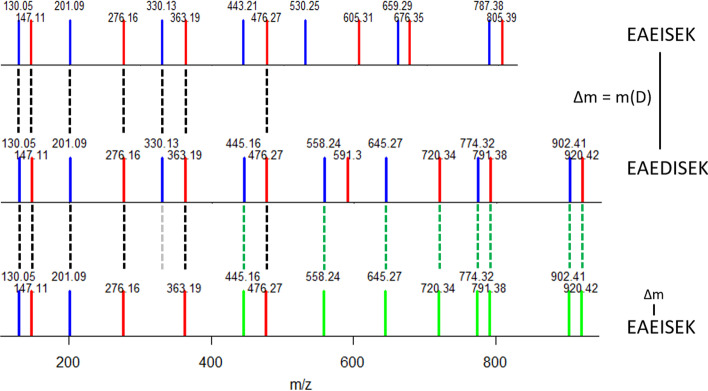
MS/MS spectra matches and their peptide sequences. *b*-ions are displayed in blue, *y*-ions in red, and matches between spectra in dashed lines. Intensities of all peaks are set to an arbitrary unit value in theoretical spectra. The EAEDISEK MS/MS spectrum (in the middle) shares 7 masses (black dashed lines) with the native EAEISEK spectrum (above). After a shift of $\Delta m$ at position 3 in EAEISEK (below), 8 new masses match with EAEDISEK (as shown in green), and one match is removed (grey dashed line). The SPC is then improved from 7 (*raw SPC*) to 14 (*shift SPC*)

Besides the comparison of spectral pairs, some methods also associate pairs of spectra when they share sequence tags [15–17]. These methods have improved the identification rate and as much as an almost twofold increase in the rate of identified spectra has been reported [15, 18]. However, despite the scientific relevance of better spectra identifications, OMS methods are still underused, notably because their reliability remains debated. It is therefore important to better describe the advantages and limitations of these methods. Because all OMS methods have in common that at some point, they must decide wether or not to use the information of $\Delta m$ within a PSM to infer a better identification, we focused our study on a thorough undertanding of two widely spread strategies to determine the best PSM for each experimental spectrum. In the first strategy (called *Strategy1* in this paper), the best PSM is chosen according to a score that does not take the $\Delta m$ into account. The second strategy (called *Strategy2*) tries to improve the alignment—and thus the score (Fig. 2)—of all the PSMs returned for a given experimental spectrum according to $\Delta m$ before the choice of the best PSM. In order to determine the most efficient strategy, a prerequisite was to be able to implement both strategies using the same software, which implies the availability of very efficient spectra comparison and alignment algorithms. The SpecOMS software [14], which we have previously developed, fulfills these conditions.

To compare in-depth the limits of each strategy, we decided to ground this study using the theoretical spectra derived from the human proteome, considering successively each theoretical spectrum in the role of an experimental spectrum. In such a way, we eliminate the inherent identification difficulties due to the imperfection of experimental spectra (noise, missing peaks, etc.) and concentrated on the benefits of each strategy. Consequently, PSMs with $\Delta m\ne 0$ can only be explained by differences in terms of amino acids, namely insertions, deletions and/or substitutions of one or several amino acids. Note that any modification (whether it is a post-translational modification (PTM) or a sequence modification) induces the same number of peak shifts in a spectrum. Every PSM matching a peptide to itself was considered irrelevant and consequently forbidden. Thus, we challenged the two above strategies to discover many diverse modifications, while keeping a certain proximity between peptides (since they arise from the same proteome). As conventional methods do, many OMS methods estimate the FDR of their results with a target/decoy approach. We also used this approach to compare both strategies, even though it is still unclear whether or not this method underestimates the incorrect identifications [19, 20]. That is why, we propose two additional measures of the PSM characteristics to evaluate their quality and compare the strategies.

## Results

We successively implemented *Strategy1* and *Strategy2* (see “Methods” section) to compare all the theoretical spectra generated from the human proteome (572,063 spectra) against a database merging the target and decoy human proteins (1,148,608 spectra). To denote unambiguously the role that each theoretical spectrum can alternately play, we call it *bait* when it plays the role of an experimental spectrum and *hit* when it represents the theoretical spectrum modeled from the protein database. By extension, we also designate by bait and hit the peptides that generated respectively these theoretical spectra. Any pair (bait,hit) having an SPC greater than or equal to our initial threshold 7 (see “Methods” section) is called a *candidate PSM*. Any PSM returned by our software will be called *best PSM*. On the one hand, *Strategy1* selects the best hit according to the *raw SPC* score, computed without taking into account the observed $\Delta m$. On the other hand, *Strategy2* iteratively tests, on each possible amino acid and at the N-terminal and C-terminal sides of the peptide, whether a modification of mass $\Delta m$ could improve the alignment between the two spectra [21]. In this latter case, a new score *shift SPC* is computed, which corresponds to the number of shared masses after realignement, and the best PSM is selected according to this new score.

**Table 1:** Number of PSMs obtained by *Strategy1* according to *raw SPC*

Min * raw SPC*	$\Delta m$ = 0		$\Delta m < { 0}$		$\Delta m > { 0}$		Total		
	#target	#decoy	#target	#decoy	#target	#decoy	#target	#decoy	FDR(%)
7	71,852	87,705	88,895	39,432	107,842	59,678	268,589	186,815	41.02
8	71,852	87,705	55,874	12,352	61,225	19,550	188,951	119,607	38.76
9	32,972	32,740	41,378	3874	40,085	4822	114,435	41,436	26.58
10	32,970	32,739	31,973	868	30,245	1026	95,188	34,633	26.68
11	10,918	11,209	26,095	310	24,547	305	61,560	11,824	16.11
12	10,918	11,208	21,553	112	20,313	94	52,784	11,414	17.78
13	2571	1180	17,893	46	16,939	34	37,403	1260	3.26
14	2571	1180	14,955	22	14,243	17	31,769	1219	3.7
15	1137	380	12,352	9	11,818	9	25,307	398	1.55
16	1137	380	10,239	6	9801	5	21,177	391	1.81
**17**	**672**	**61**	**8403**	**5**	**8085**	**4**	**17,160**	**70**	**0.41**
18	672	61	6907	4	6662	3	14,241	68	0.48
19	495	49	5590	2	5413	1	11,498	52	0.45
20	495	49	4557	1	4415	1	9467	51	0.54

The best PSMs provided by SpecOMS are separated into three groups based on $\Delta m$, and subdivided in each group by the origin of the hit (target or decoy database). An FDR < 1% is reached whenever * raw SPC* is greater than or equal to 17 (row in bold)

**Table 2:** Number of PSMs obtained by *Strategy2* according to *shift SPC*

Min *** shift SPC***	$\Delta m {= 0}$		$\Delta m < {0}$		$\Delta m > {0}$		Total		
	#target	#decoy	#target	#decoy	#target	#decoy	#target	#decoy	FDR(%)
7	14,207	17,401	145,801	86,218	122,479	69,298	282,487	17,2917	37.97
9	5132	4787	145,756	86,183	122,465	69,287	273,353	160,257	36.96
11	1944	1793	144,795	85,247	121,833	68,671	268,572	155,711	36.7
13	721	343	139,895	80,327	119,006	65,901	259,622	146,571	36.08
15	496	187	116,674	61,154	110,457	58,923	227,627	120,264	34.57
17	341	42	58,584	130,53	55,297	130,52	114,222	26,147	18.63
19	250	36	37,840	1681	35,439	1647	73,529	3364	4.37
**21**	**201**	**12**	**29,675**	**271**	**27,908**	**251**	**57,784**	**534**	**0.92**
23	160	10	24,262	87	22,881	82	47,303	179	0.38
25	131	5	20,036	35	18,910	36	39,077	76	0.19
27	108	4	16,686	18	15,792	14	32,586	36	0.11
29	92	2	13,835	12	13,120	9	27,047	23	0.08
31	67	2	11,345	4	10,801	5	22,213	11	0.05
33	54	0	9314	2	8888	3	18,256	5	0.03
35	44	0	7608	2	7296	2	14,948	4	0.03
37	39	0	6231	2	5989	2	12,259	4	0.03
39	17	0	5008	1	4832	1	9857	2	0.02

The best PSMs provided by SpecOMS are separated into three groups based on $\Delta m$, and subdivided in each group by the origin of the hit (target or decoy database). An FDR < 1% is reached whenever * shift SPC* is greater than or equal to 21 (row in bold). Only results for odd minimum * shift SPC* are displayed here: indeed, * shift SPC* is always even, since a match always involves both a *b*-ion and a *y*-ion after realignment. The results we obtain are thus the same for consecutive odd and even * shift SPC* (except for a few exceptions due to overlapping masses)

The results are summarized in Tables 1 and 2. In each of the two strategies, SpecOMS reported 455,404 best PSMs structured in the form of tuples (*bait,hit,SPC,*$\Delta m$), where (a) *hit* is the best candidate for *bait* (depending on the selected strategy), (b) *SPC* is the number of shared masses between bait and hit (according to *raw SPC* in *Strategy1* and to *shift SPC* in *Strategy2*), (c) a mass difference of $\Delta m$ (expressed in Daltons) exists between bait and hit—more precisely $\Delta m$ is equal to mass of bait minus mass of hit.

About 80% of the 572,063 tryptic peptides from the human proteome share at least 7 peaks with any other peptide, and about 23% of them share at least 10 peaks (target or decoy).

Since the number of best PSMs obtained by SpecOMS only depends on the number of pairs of spectra that initially share at least 7 masses (7 being the threshold parameter chosen in this study, see “Methods” section), the number of best PSMs remains identical in both strategies. By contrast, *Strategy1* and *Strategy2* provide dissimilar sets of best PSMs, that we respectively named $\hbox {PSM}_1$ and $\hbox {PSM}_2$ (see Fig. 6, “Methods” section). About 37% of the PSMs (167,291 baits) differ between both strategies when the initial SPC threshold is set to 7. We separated $\hbox {PSM}_1$ and $\hbox {PSM}_2$ into 3 groups according to $\Delta m$ : $G_1$ is the group of PSMs such that $\Delta m=0$ (i.e., the bait and the hit have the same mass), $G_2$ is the group such that $\Delta m>0$ (the mass of the bait exceeds the mass of the hit) and $G_3$ is the group such that $\Delta m<0$ (the mass of the hit exceeds the mass of the bait). Note that when $\Delta m\ne 0$ in *Strategy2*, the score of the corresponding PSMs is likely to increase, while the score of PSMs with $\Delta m=0$ remains unchanged (since no realignment is possible). As a result, many candidate PSMs selected with $\Delta m=0$ in *Strategy1* has been overpassed by PSMs associated to $\Delta m\ne0$ in *Strategy2* (127,949 PSMs). Next, we evaluated the confidence of the results with the typical target-decoy approach [7] and compute the minimum similarity score that guarantees a FDR usually accepted by users. Respectively to a FDR < 1%, *Strategy1* validates 17,160 PSMs with a minimal *raw SPC* of 17 (i.e. considering best PSMs for which *raw SPC* $\ge$ 17), while *Strategy2* validates 57,784 PSMs with a minimal *shift SPC* of 21 (i.e. considering best PSMs for which *shift SPC* $\ge$ 21). *Strategy2* recruits more than three times more PSMs than *Strategy1* so, we can conclude that *Strategy2* behaves better than *Strategy1* according to the number of validated PSMs. Thus, 3% to 10% of the theoretical spectra have at least one “neighbor peptide” that shares a sufficient number of masses so that their similarity is not estimated to be considered as the result of chance. It must be remembered that a hit is supposed to directly identify a bait for PSMs with $\Delta m=0$. We know that it cannot be the case in our dataset, since we set SpecOMS so as to forbid PSMs involving the same peptide. Indeed, very few PSMs were validated by the target-decoy method in $G_1$ (672 PSMS with *Strategy1*, 201 PSMs with *Strategy2*), which is consistent with the composition of this group. On the opposite, the $G_2$ and $G_3$ groups represent most of the validated PSMs, but one may wonder to what extent the information given by these PSMs is enough to restore the correct amino acid sequence of the baits—a *sine qua non* condition to consider a posteriori an identification as correct.

**Fig. 3:**
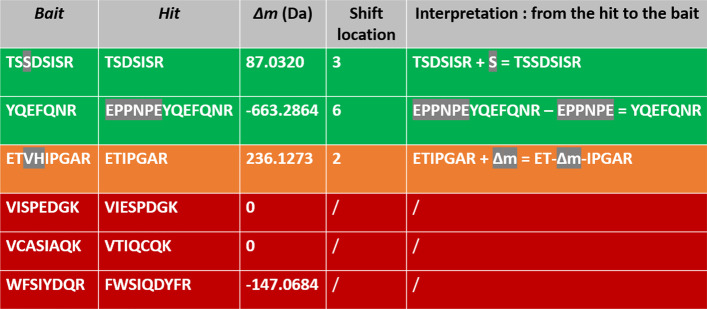
Illustration of the Green/Orange/Red classification of PSMs. The first two rows present PSMs with a bait unambiguously deductible from the information given by the PSM. Such PSMs are classified as Green. In the first example, $\Delta m$ corresponds to the mass of S, which can thus be added in the hit at the given location to retrieve the bait. In the second example, the absolute value of $\Delta m$ corresponds to the mass of EPPNPE, which can be deleted from the hit to retrieve the bait. In the third row, $\Delta m$ can correspond to two possible amino acid sequences (VH or HV). Such PSM is thus classified as Orange. In the last three rows, transforming hit into bait is more difficult because there is too much ambiguity, although sequences may be close as in the first red row. In all cases, such PSMs are classified as Red

To answer this question, we proposed new criteria to measure the quality of identifications. Knowing exactly which peptide generated each bait, we should be able to accurately assess the effectiveness of each strategy. Firstly, we defined a new classification that reflects the level of difficulty for a user or software to retrieve the bait sequence using both the hit sequence, the value of $\Delta m$ and the shift location. PSMs with $\Delta m\ne 0$ can only be explained by differences in terms of amino acid sequences. Then, by applying one or several editing operations (insertion(s), deletion(s), and/or substitution(s)), the hit can always be transformed into the bait. Given a PSM, if the bait can be deduced from the hit without any ambiguity in just one editing operation specified by $\Delta m$, we classified this PSM as Green. However, when $\Delta m$ corresponds to an insertion of several amino acids, this is not enough to reconstruct the sequence of the bait without ambiguity—several permutations of amino acids can lead to the same $\Delta m$. In this case, we classified the PSM as Orange. We classified all other PSMs as Red (see “Methods” section). As a result, the Red class contains a large variety of PSMs: those that require several to many editing operations to transform the bait into the hit (in other words, bait and hit sequences are very dissimilar), but also those whose sequences may be close, but that contain permutations of amino acids that are difficult to infer from $\Delta m$. In particular, PSMs with $\Delta m = 0$ are obviously classified as Red, because the hit and the bait are different and no realignment is possible. An illustration of the color classification containing more examples is given in Fig. 3.

Secondly, we introduced a new feature, which we call the * Low Information Peaks Rate* (LIPR), that measures, for a given PSM, the proportion of masses that are shared by two spectra but correspond to different amino acid sequences (see “Methods” section). In summary, the higher this value, the less sequence information the PSM carries, in the sense that proportionally many shared peaks correspond to distinct amino acid sequences.

**Fig. 4:**
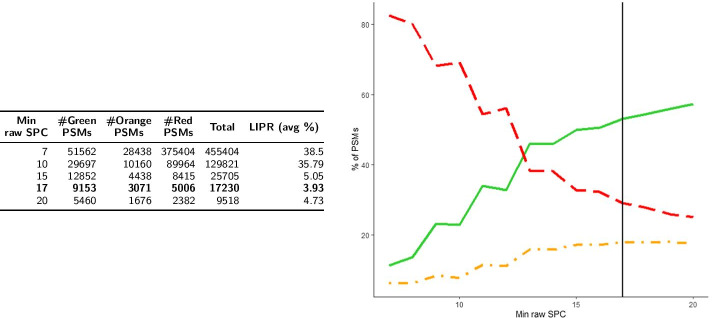
Color classification and LIPR for *Strategy1* ($\hbox {PSM}_1$). Number of PSMs in the three color categories and average LIPR according to minimum * raw SPC* (left). Percentage of PSMs in the three color categories (Green: solid line, Orange: dotted and dashed line, Red: dashed line) according to * raw SPC* (right). The vertical line is located at FDR < 1% (*raw SPC* = 17)

**Fig. 5:**
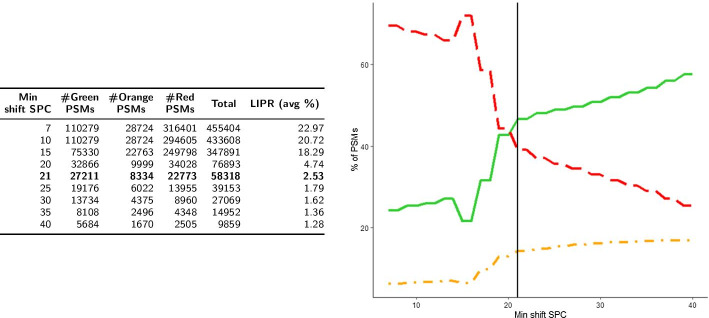
Color classification and LIPR for *Strategy2* ($\hbox {PSM}_2$). Number of PSMs in the three color categories and average LIPR according to minimum * shift SPC* (left). Percentage of PSMs in the three color categories (Green: solid line, Orange: dotted and dashed line, Red: dashed line) according to * shift SPC* (right). The vertical line is located at FDR < 1% (*shift SPC* = 21)

We present the distributions of the sets $\hbox {PSM}_1$ and $\hbox {PSM}_2$ obtained respectively by *Strategy1* and *Strategy2* among the 3 color classes, as well as the evaluation of the LIPR feature, in Figs. 4 and 5 . It can be seen that, globally, both strategies behave in a similar fashion, but at 1% FDR, *Strategy2* validates roughly three times more Green PSMs than *Strategy1* (27,211 vs 9153). Thus, at first glance, the number of additional identifications obtained by *Strategy2* (compared to *Strategy1*) does not come at the cost of a deterioration of the quality of the results.

In terms of LIPR, it can be noted that its average value is higher for $\hbox {PSM}_1$ (38.5% for $\hbox {PSM}_1$ vs 22.97% for $\hbox {PSM}_2$). Generally speaking, in both strategies the LIPR decreases when the minimum SPC increases. At a 1% FDR threshold, results are quite similar for both strategies (3.93% for $\hbox {PSM}_1$ vs 2.53% for $\hbox {PSM}_2$).

A strong difference concerning LIPR appears between target and decoy PSMs, and this phenomenon is present in both strategies (results not shown). The average LIPR of target PSMs in $\hbox {PSM}_1$ is 31%, whereas it reaches 49% in decoy PSMs. This difference increases with the minimum SPC that is observed, and the LIPR suddenly drops near the 1% FDR threshold ; for instance, for * raw SPC* $\ge$ 15 in $\hbox {PSM}_1$, the average LIPR of decoy PSMs is 64% whereas it is 4% for target PSMs. The LIPR can thus be seen as a way to capture the “randomness” of decoy PSMs, for which matches between peaks that correspond to different sequences are much more common than in target PSMs, especially for PSMs with high SPC values.

To highlight the differences between the PSMs provided by each strategy, we isolated PSMs specific to *Strategy1* (a set of PSMs called $\hbox {SS}_1$) from PSMs specific to *Strategy2* (a set of PSMs called $\hbox {SS}_2$). Since $\hbox {SS}_1$ and $\hbox {SS}_2$ are exactly what differentiates * Strategy1* from * Strategy2*, highlighting the main differences (or similarities) between these two sets is particularly informative. It should also be noted that this comparison can only be done at SPC = 7, thus with the whole set of (specific) PSMs, since * raw SPC* and * shift SPC* are by definition distinct scores.

The number of PSMs in each color class, as well as the average LIPR for $\hbox {SS}_1$ and $\hbox {SS}_2$ are displayed in Table 3. We can clearly see that *Strategy2* contains many more PSMs classified as Green, compared to *Strategy1* (more than 16 times more). The average LIPR is also much higher in $\hbox {SS}_1$ (61.7%) than in $\hbox {SS}_2$ (19.44%).

The results we obtain concerning the color classification and LIPR can be interpreted in two different manners. First, they can be considered independently of the FDR, based on the argument that the FDR is a measure that remains debated in OMS methods. Based on the results presented in Table 3, it can be seen that *Strategy2* clearly outperforms *Strategy1*. Another way to consider these two indicators is to compare them taking the FDR into account (namely, at an FDR less than 1%). In that case (see Figs. 4 and 5) if *Strategy2* still outperforms *Strategy1* in terms of number of PSMs (roughly 3 times more Green or Orange PSMs in *Strategy2* than in *Strategy1*), the results in terms of percentage are in favour of *Strategy1* (70.9% of the PSMs are Green or Orange in *Strategy1* vs 60.9% for *Strategy2*). However, the fact that our color classification marks as Red proportionally more PSMs from *Strategy2* than from *Strategy1* must be put into perspective with a lower LIPR value associated to *Strategy2*, indicating a close proximity between the bait and the hit. So, a large number of these Red PSMs are likely due to a very small number of editing operations like permutations in sequences. Then, a deeper analysis of the Red category should convert, with minimal additional computational efforts, many Red PSMs into Green or Orange PSMs. Besides, we can note the high proportion of PSMs from the human proteome classified as Green when SPC $\ge 7$. If all the hits associated to those Green PSMs came from the target database, it would be come as very good news because these PSMs refer to peptides that differ by unambiguous editing operations from their “closest neighbor” (from 11% in *Strategy1* to 24% in *Strategy2*). Since it is not the case, it means that the decoy database is not only constituted of “incorrect sequences” as it should be. Then, it is clear that for both strategies, the presence of many Green PSMs in the decoy database hinders the identification of the baits.

**Table 3:** Average LIPR and distribution of PSMs in results specific to $\hbox {PSM}_1$ ($\hbox {SS}_1$) and specific to $\hbox {PSM}_2$ ($\hbox {SS}_2$) in the three color categories

Dataset	#Green * PSMs*	#Orange * PSMs*	#Red * PSMs*	Total	LIPR (avg %)
$\hbox {SS}_1$	3858	6507	156,926	167,291	61.7
$\hbox {SS}_2$	62,575	6793	97,923	167,291	19.44

## Discussion

In this work, we aimed at comparing two OMS strategies that frequently appear in recently published OMS methods. Evaluating the respective performances of the two studied strategies has been conducted through three different measures: along with the FDR, we introduced two ways of interpreting the PSMs: the first one is a color classification of the PSMs and the second one, called LIPR, represents the ratio of common peaks that disagree in terms of sequence. In summary, all three indicators show that *Strategy2* outperforms *Strategy1*. Note that the fact that *Strategy2* outperforms *Strategy1* could seem obvious at first sight, following the idea that realigning the peaks (as done in *Strategy2*) could only lead to better results. However, since *Strategy2* naturally widens the search space for each bait spectrum—since it tries to explain $\Delta m$ at different locations in the hit spectra—, this may actually lead to numerous erroneous PSMs. Hence, the fact that *Strategy2* behaves better than *Strategy1* (with respect to the FDR in this case) was not necessarily easily predictable.

The performances obtained by *Strategy2* also lead us to conclude that, among the two, *Strategy2* is the one that should be implemented in OMS software. Spectral Alignment [5] was already based on a score involving $\Delta m$ to select the best PSM by using, similarly to *Strategy2*, a realignment process. However, it should be noted that *Strategy2* requires to realign very efficiently pairs of spectra if one wants to maintain fast execution time. More recently, MODPlus [16] and Open-pFind [15] select the set of candidate PSMs with sequence tags and then perform realignments. ANN-SoLo [10, 11] and the Hybrid search [9] approximate the improved score obtained taking modified peaks into account (ANN-SoLo’s shifted dot product, hybrid search’s cosine similarity). On the other hand, some OMS methods still rely on *Strategy1* (e.g. MSFragger [12], MetaMorpheus [13]) and do not take $\Delta m$ into account to choose the best candidate PSM—although they may use it once the best candidate is chosen in order to locate the modification. Concerning our color classification, we saw that although *Strategy2* recruits more Green and Orange PSMs than *Strategy1*, it contains proportionally more Red PSMs. However, the average LIPR from the Red class obtained by *Strategy2* is much lower than for *Strategy1*. This leads us to think that a proportion of the Red PSMs from *Strategy2*, that share enough peaks corresponding to common subsequences, could be considered as “almost valid” PSMs. More precisely, we believe that with additional methodological and computational effort, some of these Red PSMs could be transfered to the Orange or Green category, an effort that methods implementing *Strategy2* should pursue.

We have conducted our study in a “strict” environment, namely comparing theoretical spectra only, and using a score (SPC) that only takes common peaks into account. Although this could be seen as a limitation, it can also be argued that comparing theoretical spectra is an opportunity to provide an overview of the proximity of tryptic peptides extracted from the human proteome from a mass spectrometry point of view. Besides, we place ourselves in an “adversary” context, where no redundancy is allowed, and thus where no PSM corresponds to an exact sequence matching. Concerning SPC, it can be noted that it is, in any method, systematically taken into account at some level in the scoring function. In our case, SPC allowed us to be context and data independent: we consider having no particular knowledge of the dataset nor the spectra at hand, and conducted our experiments in an “unsupervised” manner. Moreover, *Strategy2* (as implemented here) always determines the exact value for our score, whereas other OMS methods use approximate scores, slightly surestimating them, which may have an impact on the results.

By comparing two OMS strategies with theoretical peptides and new indicators, we also developped an environment which allowed us to see and understand elements that are more difficult to see in an experimental context. This protocol could be used to understand principles that are at the heart of other (OMS) MS identification tools, perceive their strengths and weaknesses, in order to configure and calibrate them. For example, the idea behind the color classification is not specific to the SpecOMS software we used nor to the mass shifting implied by *Strategy2*, and could help assessing the efficiency of any OMS research tool. Furthermore, we believe that our two indicators (used here in a context where both spectra are theoretical) can be adapted—to some extent—to classical MS/MS experiments in which theoretical spectra need to be compared to experimental spectra.

Our study also calls for several possible perspectives and extensions, which we briefly mention here: apply our study to experimental contexts (which would require to adapt LIPR and color classification), use a more elaborate score than SPC, improve our color classification algorithm so as to “explain” more PSMs from the current Red class, allow more than one location to explain $\Delta m$, and finally use other types of decoy database. Any combination of the above suggestions could also be considered.

## Conclusion

Despite recent progress, much remains to be done concerning the identification of MS/MS spectra carrying modifications, and the quality evaluation of OMS methods. The OMS methods present different strategies to identify the best PSM and, in this paper, we compared two such strategies that are well represented in the recent methods. We led this study in a strictly identical environment for both strategies by implementing them in the same software (SpecOMS [14]) and on an ideal dataset formed by theoretical spectra only, that we compare to itself, in order to get rid of identification difficulties [22] due to the imperfection of experimental spectra (noise, missing peaks, etc.). In addition to the conventional FDR, we introduced two new criteria to evaluate the quality of PSMs. For each of the observed criteria, *Strategy2*, which attempts to align $\Delta m$ between spectra before choosing the best candidate PSM, has always proven to be better. Overall, *Strategy2* promotes the PSMs where the hit is derived from the bait by easily identifiable editing operations. Then, more often, the sequence of the bait can be properly determined with *Strategy2*.

We can note that, regardless of the origin, a modification has the same impact on spectra and therefore, even though we did not perform the comparison on experimental spectra, we can extrapolate the superiority of *Strategy2* in the analysis of any experimental dataset. According to these results, OMS methods should implement *Strategy2* but we have to emphasize that *Strategy2* requires an efficient algorithm to find the best alignment of spectra on a very large number of candidate PSMs, the likely reason why it is not done in all OMS methods.

We can also note that OMS methods further complicate the protein inference problem [23, 24], since the bait peptide should not be considered as known until $\Delta m$ is explained. Typically, the bait peptides with unexplained $\Delta m$ are those marked as Red in our classification. This confirms the need to investigate more deeply the Red PSMs, by providing an automatic method retrieving bait from hit in these PSMs. Generally, we showed that the color classification and also the LIPR are pertinent indicators that can be considered independently of the FDR. Then, with some additionnal efforts, it would be interesting to adapt these criteria to usual MS/MS experiments in which theoretical spectra need to be compared to experimental spectra.

## Methods

### Peptide identification using SpecOMS

We implemented two strategies to find the best PSMs using the SpecOMS software [14]. Next, we applied these two strategies to compare the large set of theoretical spectra generated from the human database against themselves. To perform this comparison between theoretical spectra, we added a new functionality to SpecOMS (in the form of a new search mode).

A peptide that plays the role of an experimental spectrum in a peptide spectrum match (PSM) is called the * bait*, whereas a peptide associated to a bait in a PSM is called a * hit*. Parameters were set in such a way that SpecOMS extracted from its data structure SpecTrees [25] all pairs of spectra of the form (bait,hit) whose shared peaks count (SPC) is greater than or equal to 7.

**Fig. 6:**
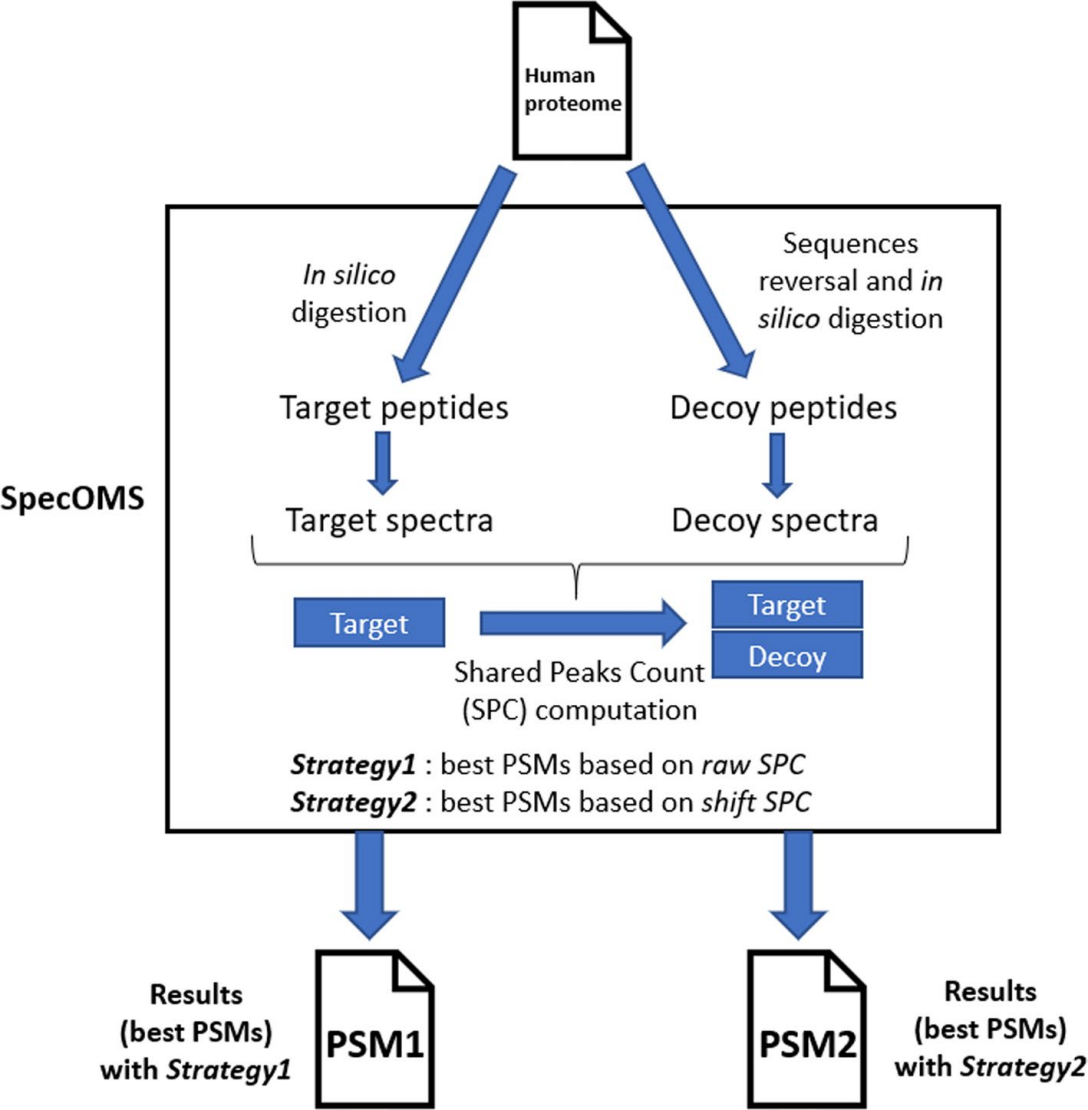
Workflow for * Strategy1* and * Strategy2*. Proteins are processed by SpecOMS in order to find the best PSM for each peptide of the database. This is done by computing the shared peaks count (SPC) between every peptide (seen as a theoretical spectrum) of the initial database, compared to all peptides (except itself) from both the target and decoy databases. In *Strategy1*, the best PSM is chosen according to the raw shared peaks count (or * raw SPC*). In *Strategy2*, an extra computation is realized for all candidate PSMs with $\Delta m\ne 0$, consisting in shifting the peaks according to $\Delta m$ at all possible locations, and keeping the best induced SPC (or * shift SPC*)

The above threshold of 7 appears to be a good trade-off for our study: first, it should not be too low so as to avoid many PSMs with a relationship carrying little information between the hit and the bait. It should not be too high either, so as to prevent the case where a low scored candidate PSM would be (definitely) discarded, since the score for such PSMs may be greatly increased (and thus may become chosen as best PSM) by our shifting procedure in *Strategy2* (see Fig. 6).

The following parameters of SpecOMS were then set in all the runs: threshold = 7 (as discussed above), single_match = true, nbMissCleavage = 0, minimumPeptideLength = 7, maximumPeptideLength = 30, maxMassesCount = 60, minimumScore = 60, decoyBase = true. Some of these parameters (and in particular minimumScore) were set so as to force SpecOMS not to search for missed cleavages nor semi-tryptic peptides: since we compare a set of peptides to itself, we know these phenomena cannot appear.

Depending on the run, the parameter “shift” of SpecOMS was set to false (*Strategy1*) or true (*Strategy2*). More precisely, in *Strategy1*, for each bait, SpecOMS selects the best PSM based on the highest SPC, a score that we call * raw SPC*.

**Fig. 7:**
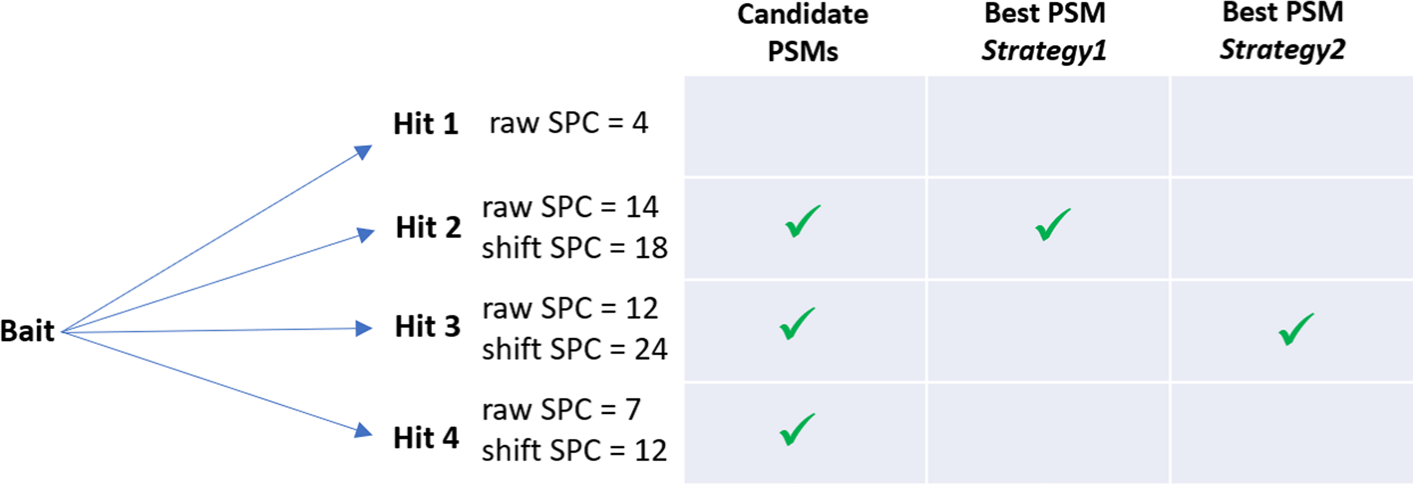
Determining the best PSM in each strategy. Suppose, fictitiously, that a given bait is to be compared to 4 peptides (called hits). Hit 1 is discarded as its * raw SPC* with bait is below the imposed threshold of 7. Hits 2, 3 and 4 are candidate PSMs for bait. If $\Delta m\ne 0$ for hits 2, 3 and 4, a shift may be applied, and in that case * shift SPC* is obtained (with * shift SPC* $\ge$ *raw SPC* by definition). In *Strategy1*, the best PSM for bait is hit 2, as it is based on * raw SPC*. In *Strategy2*, the best PSM for bait is hit 3, as it is based on * shift SPC*

In *Strategy2*, the best PSM for a given bait *b* is selected after the following two-step procedure is applied: first, for every candidate hit *h* for *b* such that $\Delta m\ne 0$, SpecOMS realigns *h* to *b* by shifting its masses (by $\Delta m$) at each possible relevant location in the spectrum, and retains the shift location in *h* that yields the best newly computed SPC (a principle frequently used to explain $\Delta m$ [26]). Second, SpecOMS chooses the best PSM among the candidate PSMs for *b*, based on the newly computed SPC, that we call * shift SPC*. See Figs. 6 and 7 for an illustration.

### Data

The human proteome was downloaded from Ensembl 99, release GrCh38 [27] on the Ensembl FTP server. Proteins predicted with the annotation “protein coding” were added to 116 contaminant proteins downloaded from the cRAP contaminant database. The resulting set of proteins is referred to as the * target* database. After in silico digestion by trypsin, which cleaves after lysine and arginine (K and R), peptides whose lengths are out of the range 7 to 30 amino acids (inclusive), together with those containing the letter ‘X’ (representing an unknown amino acid—about 3%) were removed. SpecOMS generated the decoy database from the target database by reversing the initial protein sequences before the *in silico* digestion is applied.

### Theoretical spectra generation

Each peptide is fragmented in silico by SpecOMS, so as to transform it into a * theoretical spectrum*. For this, ions from the *b* and *y* series are generated, each with the same intensity unit, as intensity is not taken into account in our study (only the shared peaks count (SPC), i.e. the number of common peaks—or masses—will be considered). For a given peptide, the set of generated masses represents its theoretical spectrum.

### Measuring the quality of PSMs

Different measures were performed on the two datasets obtained with *Strategy1* or *Strategy2*, in particular to determine to which extent the chosen identification strategy impacts the results.

The first classical measure that we used is the number of PSMs we can validate at a given False Discovery Rate (FDR). We calculated the FDR as the proportion of best PSMs of the form (bait,hit) for which the hit is a decoy, over the total number of best PSMs. In this work, we were essentially interested by PSMs for which the FDR is less than 1%.

#### The Green/Orange/Red classification

Another parameter which we consider as informative for validating MS/MS results, notably in this context, is our ability to explain a PSM of the form (bait,hit) obtained by a given strategy ; by “explain”, we mean unambiguously determine the transformation (in terms of amino acid sequence) that is required to retrieve the bait starting from the hit. Recall that since distinct pairwise peptides are compared, bait and hit necessarily differ in terms of sequence ; besides, they cannot differ otherwise (e.g. due to chemical modifications) since our set is composed of theoretical spectra only. Thus, the question we ask ourselves is the following: given a PSM (bait,hit) together with $\Delta m$, * shift SPC* and its correspondng best shift location, how difficult is it to precisely explain bait from hit ? For this, we introduce here a classification of PSMs into three colors (Green, Orange or Red), depending on this level of difficulty, from the easiest (Green) to the hardest (Red). In a nutshell, Green means that we are able to explain the link between hit and bait unambiguously, Orange contains some level of ambiguity, and Red means that further information and/or computational efforts are necessary to explain the relationship between bait and hit. For instance, if $\Delta m$ is explained by a single insertion, deletion or substitution, the corresponding PSM will be classified as Green, whereas if several consecutive insertions, deletions and/or substitutions are necessary at a given location, it will be classified as Orange since the location is known, but the sequence order is not completely determined (e.g., if we know that amino acids A and L need to be inserted at a given location, some ambiguity remains since we can either insert “AL” or “LA”). Finally, a PSM is Red whenever it is neither Green or Orange. Red thus represents either valid identifications that are too difficult to explain (e.g., when bait and hit differ at two or more distinct locations), or invalid ones (e.g., when $\Delta m=0$, while bait and hit have very dissimilar sequences). Algorithm 1 describes our classification algorithm in details, and we also refer to Fig. 3 for an illustration on different examples.

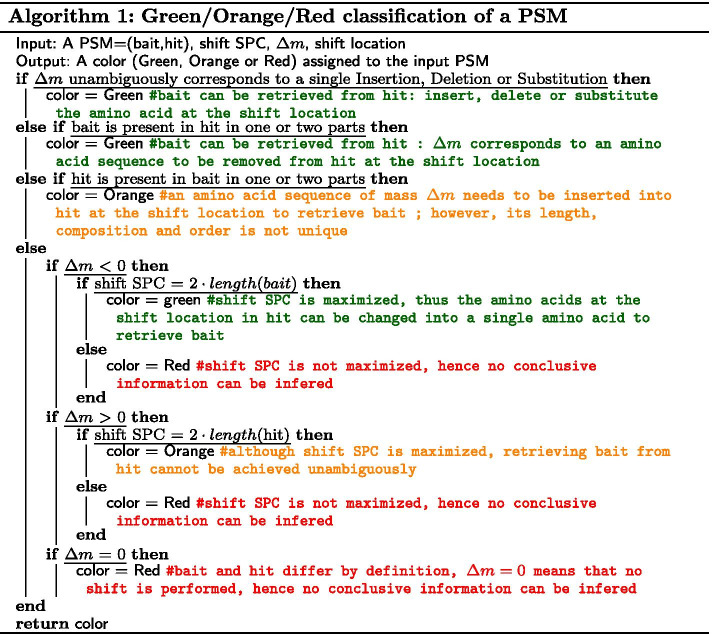



#### Low Information Peaks Rate (LIPR)

In an MS/MS experiment, spectra are considered similar to each other if they share a high number of masses. Ions from the same series (i.e., *y*-ions or *b*-ions in our case), which represent the same fragment, necessarily possess the same mass. Consequently, common masses represent relevant information concerning sequence similarity. However, the converse is not always true: identical masses may not represent identical sequences, for example when amino acids are permuted (e.g., AEAE and EEAA have the same mass) or in more complex situations when combinations of different amino acids turn out to have the same total mass (e.g., KE and GVT have the same mass).

**Fig. 8:**
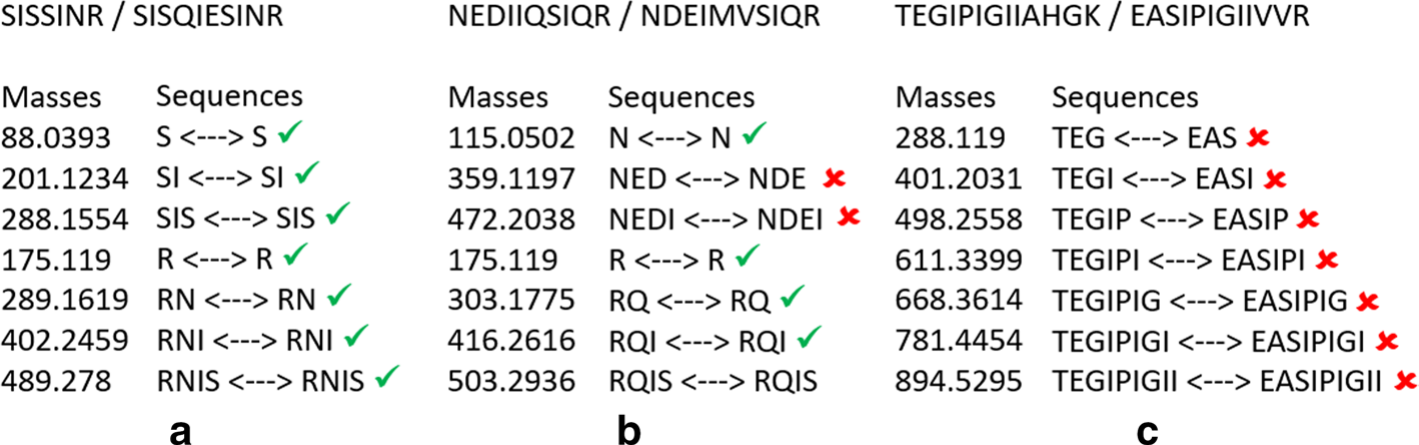
Low Information Peaks Rate (LIPR) for 3 different PSMs. Masses are in Dalton, and the “Sequences” column in each example represents all fragmented peptides having the same mass for the considered PSM. A green check symbol denotes similar sequences, whereas a red cross denotes distinct sequences. **a** No common masses corresponds to distinct amino acid sequences, thus the LIPR for this PSM is equal to 0%; **b** 2 common masses over a total of 7 correspond to distinct sequences, thus the LIPR for this PSM is equal to $\frac{2}{7}=28.57\%$; **c** all common masses in this PSM correspond to distinct sequences, hence the LIPR for this PSM is equal to 100%

Following the above discussion, we introduce here a new measure, that we call Low Information Peaks Rate (or LIPR), which is defined as follows: for a given PSM (bait,hit), LIPR(bait,hit) is the ratio of common masses between bait and hit that * do not* correspond to identical sequences (see Fig. 8 for an illustration). LIPR is thus a value between 0 and 1 (which we will express as a percentage) ; a LIPR close to 0 implies that the two amino acid sequences of bait and hit are very similar. In that case, one can argue that the PSM at hand is relevant, and that retrieving bait from hit may be feasible. On the other hand, when LIPR is close to 1, both sequences, although sharing a non negligible number of masses, represent very dissimilar sequences, and the PSM can thus be considered as debatable.

## Data Availability

The human proteome was downloaded from Ensembl 99, release GrCh38 on the Ensembl FTP server ftp://ftp.ensembl.org/pub/release-99/fasta/homo sapiens/pep/. Proteins predicted with the annotation “protein coding” were added to 116 contaminant proteins downloaded from the cRAP contaminant database ftp://ftp.thegpm.org/fasta/cRAP. The SpecOMS software is available at https://github.com/dominique-tessier/SpecOMS.

## Abbreviations

FDR: False discovery rate
LIPR: Low information peaks rate
MS: Mass spectrometry
OMS: Open Modification Search
PSM: Peptide spectrum match
PTM: Post-translational modification
SPC: Shared peaks count
